# Substrate and Plant Genotype Strongly Influence the Growth and Gene Expression Response to *Trichoderma afroharzianum* T22 in Sugar Beet

**DOI:** 10.3390/plants9081005

**Published:** 2020-08-07

**Authors:** John Schmidt, Bradley R. Dotson, Ludwig Schmiderer, Adriaan van Tour, Banushree Kumar, Salla Marttila, Kenneth M. Fredlund, Susanne Widell, Allan G. Rasmusson

**Affiliations:** 1 Department of Biology, Lund University, Sölvegatan 35B, SE–223 62 Lund, Sweden; john--schmidt@hotmail.com (J.S.); bradley_robert.dotson@biol.lu.se (B.R.D.); ludwig.schmiderer@gmail.com (L.S.); adriaan.vantour@gmail.com (A.v.T.); banu.kumar91@gmail.com (B.K.); susanne.widell@biol.lu.se (S.W.); 2 MariboHilleshög AB, Säbyholmsv. 24, 261 91 Landskrona, Sweden; kenneth.fredlund@carlsberg.com; 3 Department of Plant Protection Biology, Swedish University of Agricultural Sciences, Box 102, SE–23053 Alnarp, Sweden; Salla.Marttila@slu.se

**Keywords:** *Beta vulgaris*, gene expression, growth stimulation, inbred genotypes, pathogen response pathways, *Trichoderma afroharzianum*

## Abstract

Many strains of *Trichoderma* fungi have beneficial effects on plant growth and pathogen control, but little is known about the importance of plant genotype, nor the underlying mechanisms. We aimed to determine the effect of sugar beet genotypic variation on *Trichoderma* biostimulation. The effect of *Trichoderma afroharzianum* T22 on sugar beet inbred genotypes were investigated in soil and on sterile agar medium regarding plant growth, and by quantitative reverse transcriptase-linked polymerase chain reaction (qRT-PCR) analysis for gene expression. In soil, T22 application induced up to 30% increase or decrease in biomass, depending on plant genotype. In contrast, T22 treatment of sterile-grown seedlings resulted in a general decrease in fresh weight and root length across all sugar beet genotypes. Root colonization of T22 did not vary between the sugar beet genotypes. Sand- and sterile-grown roots were investigated by qRT-PCR for expression of marker genes for pathogen response pathways. Genotype-dependent effects of T22 on, especially, the jasmonic acid/ethylene expression marker *PR3* were observed, and the effects were further dependent on the growth system used. Thus, both growth substrate and sugar beet genotype strongly affect the outcome of inoculation with *T. afroharzianum* T22.

## 1. Introduction

*Trichoderma* spp. are filamentous fungi that are commonly found in soil, rhizosphere, and foliar environments, and some species have been found to grow endophytically [1,2]. Strains of several species are beneficial to plants. They are used for both growth promotion and as biocontrol agents against a broad variety of diseases in crops, including cereals, oil seeds, and even trees [3,4,5,6]. Substantial growth promotion by *Trichoderma harzianum* strains has been observed for a number of horticultural species, such as petunia, chrysanthemum, tomato, and red pepper, including an up to 80% increase in root area and dry weight (DW) in cucumber seedlings [7,8]. *Trichoderma* can exert beneficial effects to plants by a combination of several mechanisms, including: competition with pathogens for space and nutrients, direct antibiotic damage to the pathogens (e.g., as part of mycoparasitism), eliciting plant pathogen defense systems, and directly stimulating plant growth and nutrient uptake [5,9]. Most of these interactions will demand active participation of both the plant and the *Trichoderma*. The direct fungal attack on pathogens involves secretion of enzymes and metabolites, including peptaibols that are membrane-permeabilizing peptides [10]. Sterile-grown plant cells have been shown susceptible to permeabilization by the model peptaibol alamethicin [11] from *Trichoderma viride* (reidentified as *T. arundinaceum*) [12]. However, pre-exposure to *Trichoderma* cellulase elicits the plant cell to modify its plasma membrane, rendering it alamethicin-resistant [13,14]. Similarly, the direct interaction between roots and *Trichoderma* hyphae involves local plant formation of novel structural and metabolic components, plant recognition of the fungal elicitors, the global orchestration of plant pathogen defense, especially via jasmonic acid (JA) and ethylene (ET) response pathways, and modification of the plant nutrient uptake processes and hormonal systems for growth regulation [3,5,15]. Examples of plant growth promotion cases investigated include the interaction of *Trichoderma afroharzianum* T22 and *Trichoderma asperelloides* T203 (both previously called *Trichoderma harzianum*) with maize and cucumber, respectively [8,16,17,18,19], and the interaction of *Trichoderma virens* and *Trichoderma atroviride* with *Arabidopsis thaliana* [20]. In the most studied system, the interaction of T22 and maize, the growth effects are, however, variable, indicating an incomplete understanding of the factors governing the outcome for the plant [5]. Also, tomato and lentil cultivars have displayed variable growth stimulation and inhibition by *Trichoderma* [21,22]. Overall, the present knowledge indicates that a large number of plant functions will be important for the outcome of plant-*Trichoderma* co-cultivation.

Sugar beet (*Beta vulgaris*) is an important crop, which is related to the crops quinoa and spinach within the amaranth family. It is relatively salt and drought tolerant, probably due to its origin from the beach plant *Beta maritima*, yet is subject to a range of diseases [23]. The diploid genome has been sequenced and found to be relatively small for a crop [24], making sugar beet an attractive species for biomolecular investigations. Research on *Trichoderma* effects on sugar beet has focused on describing induced resistance to pathogens that are common in sugar beet agriculture. Field trials have shown an increased resistance to leaf spot disease, caused by the fungus *Cercospora beticola,* and amelioration of damping-off and root rot infections after *Trichoderma* spp. treatment [25,26,27]. However, the direct effect of *Trichoderma* on growth in sugar beet has not been studied.

As an initial step for characterizing sugar beet–*Trichoderma* interaction, this work investigated whether there is a variable effect of the commonly used single strain biocontrol and biostimulant agent *T. afroharzianum* T22 on inbred genotypes of sugar beet, and if gene expression in response to T22 differs between genotypes. We report a pronounced effect of the sugar beet genome on the outcome of the interaction, including both stimulation and inhibition of plant growth, and that absolute changes also depended on the plant growth systems used. Two lines, with different growth responses to T22, were further shown to have a pronounced variation in gene expression responses for marker genes for different pathogen response pathways. Similar to growth outcome, the gene responses also depend on the plant growth substrate used.

## 2. Results

### 2.1. Presence of Trichoderma Promotes or Decreases Sugar Beet Growth in Soil

**Figure 1:**
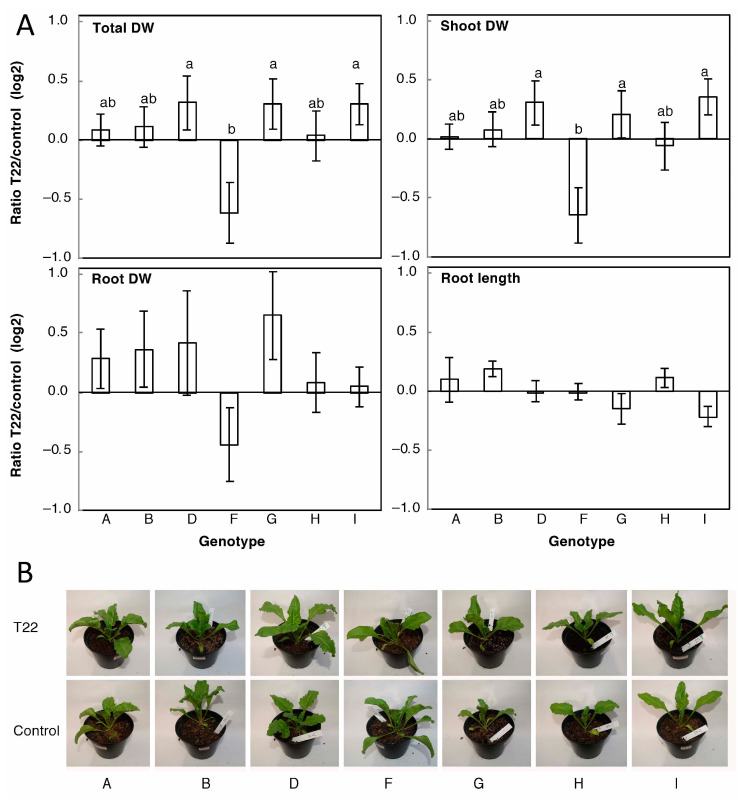
Effect of T22 on the growth of inbred sugar beet genotypes in soil. Twenty-day-old plants were treated with T22 or water, and grown in parallel for 2 or 3 weeks in two separate experiments. (**A**) shows the average effect of T22 ± SE (*n* = 9–12 plants) for both experiments pooled. The average total fresh weight and dry weight (DW) at harvest were 9.0 ± 0.4 and 0.71 ± 0.03 g, respectively. Significant differences are denoted by different letters. In (**B**), representative plants from selected lines 2 weeks after treatment are shown.

Seven inbred sugar beet genotypes, representing a wide variation in susceptibility to various pathogens, were grown in soil and treated with *T. afroharzianum* T22 conidiospores. A general trend of increased total, shoot, and root biomass accumulation was observed (Figure 1A), but without an obvious difference in plant vegetative morphology (Figure 1B). However, a 1.8-fold difference (0.85 in log2 units) between genotypes in the effect of T22 on the total DW was observed. Specifically, one line, designated F, displayed a negative effect by T22 treatment on total and shoot DW, and deviated significantly from three genotypes (D, G, and I), which were positively affected by T22. Three genotypes showed no tendency to be affected by T22. Root DW displayed a similar trend as shoot and total DW, though differences were not significant. Root length was unaffected by T22 (Figure 1). T22 also had no effect (less than 13%) on shoot to root DW ratio in any of the lines.

### 2.2. Roots of Genotype A and F Show a Difference in Gene Response to T22

For investigating potential differences in gene responses, we grew sugar beet seedlings of lines A and F on a sand/loam mix and treated with germinated T22 conidiospores for 8 h. The sand/loam mix was chosen because it could be rapidly removed by washing (<30 s per plant), allowing RNA isolation from relatively unperturbed roots without contaminating soil. Sugar beet orthologues to marker genes for different pathogen response pathways characterized in *A. thaliana* [28,29] were identified by two-way BLASTP searches (Table 1). The investigated genes included markers for the salicylic acid (SA) pathway (*PATHOGENESIS-RELATED1; PR1*), and genes associated with the ET (*RELATED TO AP2–3*; *RAP2–3*), JA (*LIPOXYGENASE2; LOX2*), and JA/ET (*PATHOGENESIS-RELATED3/BASIC CHITINASE; PR3*/*CHIB*) pathways.

**Figure 2:**
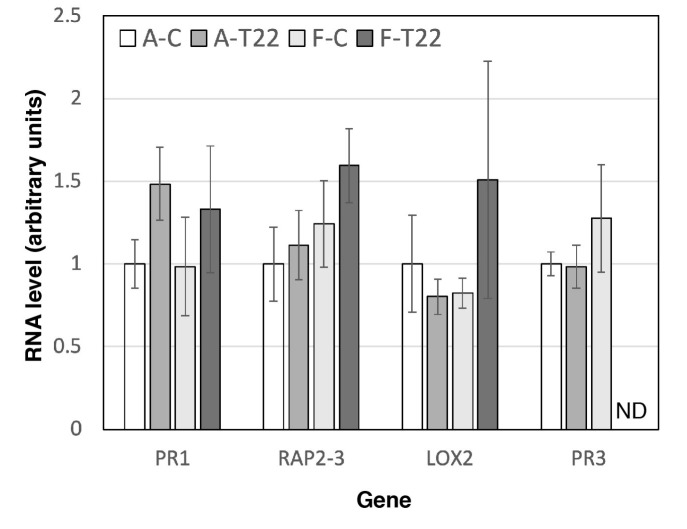
Gene responses to T22 treatment. Transcript levels in sand-grown roots of genotypes A (A-C, A-T22) and F (F-C, F-T22) are shown at 8 h after treatment with germinated T22 conidiospores (A-T22, F-T22) and water control (A-C, F-C). Data are presented as averages ± SE for three biological replicates, and ND denotes not detected. The complete disappearance of the PR3 transcript upon T22-treatment of genotype F was confirmed by agarose gel analysis, where no amplified band could be found present.

Genotypes A and F were found to have similar transcript levels for all four pathogenesis-associated genes under control conditions (Figure 2). For most of the genes, treatment with T22 did not induce changes; minor upwards trends (27–60%) for *PR1* in line A and *PR1*, *RAP2–*3, and *LOX2* in line F were not significant. However, whereas genotype A maintained *PR3* mRNA levels similar to the control also after T22 treatment, the *PR3* transcript disappeared after T22 treatment of genotype F (Figure 2). The transcript could no longer be detected by real-time or by endpoint RT-PCR analysis.

### 2.3. T22 Decreases the Growth of Sugar Beet Seedlings in Sterile Culture

**Figure 3:**
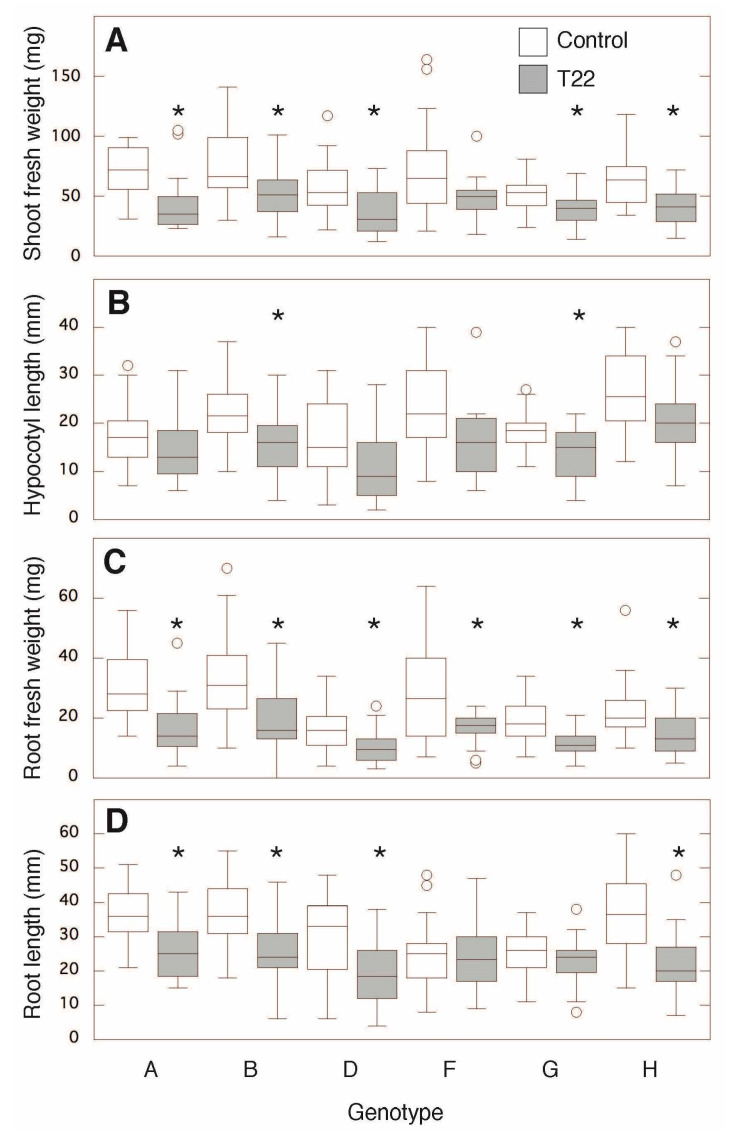
Effects of T22 on sugar beet seedling growth under sterile conditions. Box and whiskers plots show the effects on (**A**) shoot FW, (**B**) hypocotyl length, (**C**) root FW and (**D**) root length by *Trichoderma afroharzianum* T22 on six genotypes of sugar beet seedlings 9 days after T22 application. Sugar beet seedlings treated with T22 and the water control are represented by filled and open boxes, respectively, and *n* is 18–24 seedlings. Significant differences between T22 and the corresponding control are denoted with asterisks, outliers are depicted as circles.

To study the plant–fungal interaction in isolation, we grew sugar beet seedlings sterile on agar and treated with T22 by root neck application of germinated conidiospores. Generally, across the genotypes, a negative effect of T22 was observed for the fresh weight (FW) of roots and shoots and for lengths of root and hypocotyl (Figure 3). The T22-induced inhibition of leaf and root FW was significant in most lines, and quantitatively varying between approximately 30% (line F) and 50% (line A). Root length was lowered by 30–50%, except for in two lines (F and G) whose root lengths were unaffected (Figure 3). Hypocotyl length was significantly decreased in genotypes B and G.

### 2.4. Genotypes A and F Are Similarly Colonised by T22

**Figure 4:**
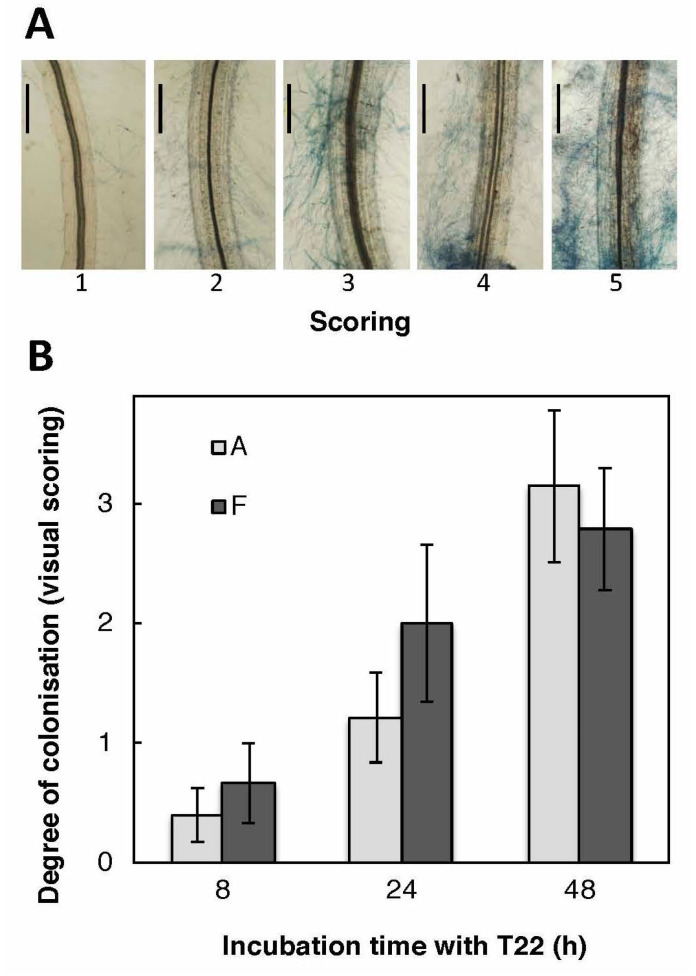
Colonization of sugar beet roots by T22. (**A**) After 48 h of co-cultivation with T22, roots were scored (1–5) for hyphae remaining adhering to the roots after the removal of the agar gel. Score 0 means lack of detectable hyphae on the roots. The bar corresponds to 200 µm. (**B**) The degree of colonization of sterile-grown sugar beet roots of genotypes A (light grey) and F (dark grey) at different time points after infection with T22. Images from lactophenol blue-stained roots were collected from early root hair zone, late root hair zone, and root base from 3–4 roots for each time point and genotype. Average scoring across all three zones ± SE (*n* = 6–7 seedlings) is shown. No colonization was observed at root tips.

Differences in T22 effects on sugar beet genotypes may be caused by different abilities for the roots to harbor T22. We therefore investigated root colonization by visual estimation of adhering hyphae. The sugar beet lines A and F were analyzed in the sterile growth system over 8–48 h after treatment with germinated conidiospores of T22. Fungal hyphae were visible in all parts of the roots (Figure 4), except for the growing root tip, and the presence of hyphae increased during the time of co-cultivation. However, the two genotypes were colonized to a similar extent by the T22.

### 2.5. Sugar Beet Genotypes Show Major Differences in Gene Expression Responses Upon Sterile Co-Cultivation with T22

**Figure 5:**
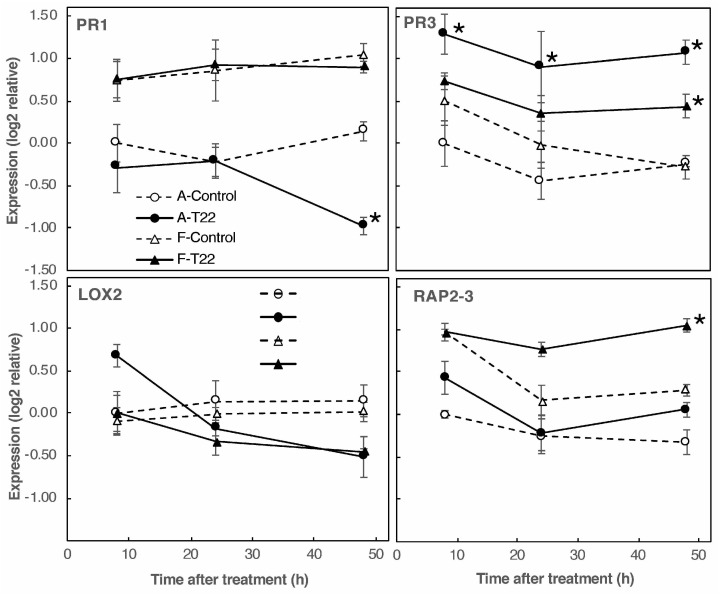
Gene responses to T22 treatment in sterile culture. Transcript levels for control and T22-treated sterile-grown roots of genotypes A and F are shown. Data are presented as average expression ± SE for 3–4 biological replicates. Values are standardized to line A controls 8 h, which were given the value zero. Significant differences between T22 treatment and control are denoted with asterisks.

We extracted total RNA from roots of sterile-grown seedlings 8–48 h after root neck treatment with germinated T22 conidiospores or water control. A major genotype-specific difference in gene expression responses was observed in the sugar beet seedlings. Genotype A displayed a rapid and significant upregulation of *PR3* and a slower downregulation of *PR1*, leading to a more than a 5-fold change in the *PR3* to *PR1* ratio 48 h after the application of T22 (Figure 5). In contrast, genotype F displayed a small and slow increase in *PR3* expression in T22-treated roots as compared to the control (circa 50% over 48 h), and no decrease in *PR1*, though the control expression was higher in line F than line A (*p* < 10^−4^). Furthermore, a small and slow decrease in *RAP2–3* (circa 50%; *p* < 10^−3^) was observed in control but not T22-treated genotype F, whereas line A was unaffected.

The results thus show that in sterile culture, roots of the two sugar beet genotypes have pronounced differences in the expressional changes of pathogen response genes induced by the presence of *T. afroharzianum* T22.

## 3. Discussion

A large number of *Trichoderma* species occur naturally in soils, and colonize roots of both monocot and eudicot plants [2]. Application of particular *Trichoderma* strains offers pathogen protection and will frequently also improve growth and performance of both healthy plants and plants under biotic or abiotic stress [1,3,5,6,9,28,31]. However, a variation between plant genotypes in the growth effect triggered by T22 has been observed, e.g., in maize. Specifically, one maize genotype showed seed yield reductions of up to 7% by T22 application in field trials [5]. Another genotype displayed up to a twofold increase in root and shoot length, although biomass accumulation was not specified [16]. In tomato, four inbred cultivars were compared for the growth effect of T22 and *Trichoderma atroviride* P1 [21]. Positive and negative effects on biomass accumulation were observed, and T22 and P1 affected individual tomato lines differently, with up to twofold differences in the T22 effect on shoot or root DW being observed.

We here found that treating soil-grown sugar beet plants with T22 affected growth (DW accumulation) of shoot and root (Figure 1), in agreement with previously published results on *Trichoderma*-enhanced plant growth in other plant species [5,7]. The response in sugar beet varied 1.8-fold between the different inbred genotypes, with one line (F) being growth-inhibited and markedly varying from three stimulated lines, and three lines being unaffected. In contrast to maize [5], the variation in the T22 effect on sugar beet biomass was not reflected in root length effects (Figure 1), suggesting instead a change in root architecture in the sugar beet. *Trichoderma* effects on DW accumulation in tomato were not coupled to changes in root length [21], and a similar case was seen in the sugar beet data (Figure 1). However, whereas *Trichoderma* induced different, or even reverse, effects on the shoot and root DW in individual tomato lines [21], the sugar beet data presented here (Figure 1) indicate similar T22-effects on shoot and root. Taken together, the variation in growth response to *Trichoderma* between plant genotypes that was earlier described for maize and tomato was also observed in sugar beet, but the patterns of response to *Trichoderma* differ between the plant species, and the negative effect in genotype F is stronger than previously reported. Putative mechanisms for decreased plant growth include cellular damage by peptaibols [11,14] or hydrolytic enzymes [32] released by *Trichoderma*, adverse effects of hormone-mimicking substances [33], or resource allocations to pathogen response pathways induced by the *Trichoderma* priming of plant immunity [34,35], and it is presently not possible to distinguish between these factors.

The above results were obtained using non-sterile growth systems that, besides *Trichoderma* and the plant, will contain various other microorganisms, even if using steam-treated soil. The growth effects could therefore be indirect and reflect *Trichoderma* effects on other microorganisms. This might be a reason why soil-grown sugar beet genotypes were generally stimulated by T22 (Figure 1), whereas sterile-grown sugar beet was mainly inhibited (Figure 3). Independent of the direction of the effects, however, the sterile system confirmed the major conclusion from the soil system, i.e., that the genotypes differed in their response to T22.

Sterile conditions have also been used when co-cultivating *Arabidopsis*, tomato, and cucumber with *Trichoderma* spp. [20,30,36]. In these experiments, growth promotions were found, again showing the variability of responses in different systems. Besides the different plant species and *Trichoderma* strains used, which may explain much of the variability, the inoculation methods cannot be neglected. In our investigations, a germinated conidiospore suspension was added directly to the root neck to allow analyses at early and defined time points after application, whereas in work by others, *Trichoderma* usually was applied at some distance below the growing root tip, also allowing plant–fungal interaction via growth-stimulating volatiles, as previously suggested [37]. In the sterile growth experiments, a small inoculum of 10–20 colony-forming units (cfu) per seedling was used and shown to be efficient when applied directly to the root neck (Figure 3). Direct application of similar numbers of cfu of *T. atroviride* has been shown to affect gene expression in *Arabidopsis*, but growth rates were not reported [38].

The presence of pathogens and beneficial microbes can induce mechanisms such as systemic acquired resistance (SAR) or induced systemic resistance (ISR). Changes in SA, JA, and ET response pathways upon *Trichoderma* treatment have been observed in multiple plant species, including, for example, *A. thaliana*, tomato, melon, cucumber, and grapevines [17,21,28,35,39,40]. In sugar beet (Figure 5), orthologues to *A. thaliana* markers for the SA (*PR1*), ET (*RAP2–3*), JA (*LOX2*), and JA/ET (*PR3*/*CHIB*) response pathways displayed variant T22-responses in two genotypes, and also depended on the plant growth substrate used. Specifically, the increase in the *PR3* chitinase gene in genotype A after treatment with T22 indicates a rapid activation of the JA/ET pathway, which is lacking in T22-treated genotype F (Figure 5). In sand-grown plants, the *PR3* transcript is unchanged by T22 in genotype A, yet disappears completely in genotype F (Figure 2). Thus, the relative T22 effect on the expression of *PR3* is relatively similar in the plant genotypes (i.e., after T22 treatment, the *PR3* expression is for both growth substrates higher in genotype A than in genotype F), yet the overall orientation of the signal changes differs between the two growth substrates. In conjunction with the opposite T22 effects on growth (Figure 1 and Figure 3), the observed gene expression changes indicate that T22 has a stronger damaging effect on the plants in the sterile system. Regarding growth effects, a similar case of damage in sterile growth systems has been observed in the sugar beet relative *Chenopodium quinoa* [37]. *Trichoderma* applied to soil for two months induced both SA and JA marker genes in tomato leaves, but relatively little in the most growth-promoted genotypes [21], consistent with a pattern where the pathogen response genes are most highly expressed under conditions where plants are little stimulated, or even inhibited, by *Trichoderma*.

*PR1* and *PR3* are both antifungal proteins and for both, examples of enhanced pathogen resistance upon transgenic transfer between plant species have been reported [41]. Thus, changes in the expression of these genes (Figure 2 and Figure 5) may be involved in the different growth responses to T22 displayed by the sugar beet lines (Figure 1 and Figure 3). Since *PR1* and *PR3* are also reporters for the SA and JA/ET pathways, respectively [41], associated response genes may also produce a differential effect. *PR3* expression depends on the action of several other response genes, and SA markedly inhibits it [41,42,43]. These mechanisms may have a part in causing the shifts observed in *PR3* and *PR1* (Figure 2 and Figure 5), consistent with the generally observed antagonism between SA and JA/ET pathways in regulating SAR and ISR responses to pathogens [3,29]. Differential effects have also been reported for beneficial microbes, e.g., application of *T. asperelloides* T203 to roots of sterile-grown cucumber seedlings induced root and leaf expression of genes involved in JA/ET signaling without affecting SA [17]. Consistently, inoculation of tomato seedlings with spores of *T. virens* TriV_JSB100 displayed a specific induction of the JA marker gene *PDF1*, similarly in two cultivars [44]. However, treatment of *A. thaliana* sterile-grown seedlings with *T. virens* induced both SA and JA marker gene reporter constructs over an 8-day period in leaves [45], as has also been observed with other growth-promoting soil microorganisms, such as *Bacillus cereus* AR156 [46]. In contrast, treatment of *A. thaliana* roots with *T. hamatum* T382 activated SA-induced genes in leaves after 2 days, but JA/ET pathway genes only after multiple treatments [35]. The differences in responses between plant species are, however, confounded by experimental variations in time of treatment and analysis. For example, *T. atroviride*-induced responses of SA, JA, and ET markers in tomato vary over time [47], and can even reciprocally change direction of induction and suppression during treatment [48]. SA, JA, and ET pathway sensitivity may also differ between roots and leaves, and be affected by the site of microbial application [49,50]. Especially, transcriptional changes in roots have been little analyzed, and then only in sterile systems, probably due to the technical difficulties of analyzing roots in soil. The observations presented here, where sugar beet growth and root gene expression responses to *Trichoderma* are highly dependent on both growth substrate and plant genotype, is a step forward towards explaining the variation observed in *Trichoderma* effects, and emphasize a need for more strict and natural experimental systems.

Several plant processes are likely involved in governing the stimulation and inhibition of plant growth. Genotypes with contrasting responses may upon molecular analyses allow mechanistic characterization of the response to *Trichoderma*. This may also lay the ground for crop breeding to enhance *Trichoderma* stimulation, thus avoiding the variability in growth response that has been found for different plants and growth conditions.

## 4. Materials and Methods

### 4.1. Biological Material

A set of sugar beet inbred genotypes (MariboHilleshög AB, Landskrona, Sweden) were selected to constitute a diversity in pathogen resistance and sensitivity, and were given a random identification letter. After excluding lines with low germination or slow or aberrant growth, seven genotypes were included in soil experiments. Preferences for the genotypes used, according to Hilleshög AB, were as follows; Genotype A is susceptible to plant pathogens, especially to *Fusarium* spp.; Genotype B is mildly resistant to *Aphanomyces* spp.; Genotype D is resistant to *Aphanomyces* spp.; Genotype F is susceptible to beet yellows virus; Genotype G is generally susceptible to plant pathogens; Genotype H is resistant to Rhizomania and *Cercospora* spp.; Genotype I is resistant to Rhizomania.

*Trichoderma afroharzianum* T22 (Rifai; ATCC20847) was obtained from ATCC (Manassas, VA, USA) and grown and maintained on potato dextrose agar, according to instructions (ATCC). For obtaining conidiospore suspensions, sterile distilled water was added to 2-week-old sporulating cultures of *T. afroharzianum* T22. The suspension was gently scraped from the agar surface, filtered through a cotton ball, and washed twice by centrifugation (2850 g for 5 min in 50 mL tubes in a table-top swing-out centrifuge) and resuspension in water. Conidiospores were germinated by incubation in a synthetic medium [51] for 18 h at 30 °C with 150 rpm shaking in a TR−225 orbital shaker (Infors, Bottmingen, Switzerland). The germinated conidiospores were washed and resuspended in water as above, and then directly used for inoculation. The procedure was carried out at 25 °C.

### 4.2. Greenhouse Growth

Sugar beet fruits, including pericarp, were consecutively washed in tap water for 30 min each at 25, 55, and 25 °C and dried on paper towels overnight. They were then sown individually in polystyrene cultivation trays containing steamed potting soil and allowed to grow in a greenhouse with supplemental metal halide light (Osram Powerstar HQI-BT, 16 h/day; approximately 150 µmol m^−2^ s^−1^) for 12 days at 23 °C. The plants were transplanted to pots (one plant per pot) and further grown under the same conditions. Twenty days after sowing, 2.5 mL of T22 conidiospore suspension that contained 2.5 × 10^6^ colony-forming units (cfu), or 2.5 mL of water (control) was added to the root neck (i.e., the hypocotyl/root transition zone) of each plant. Genotypes were grown in a diagonal setup of the pots with rotation three times per week. Root lengths were determined after rinsing in water, and DW after drying tissues for 40 h at 70 °C. Damages to minor roots upon extraction from soil were assumed not to bias weight determinations. Data points deviating more than 3 SDs from the average of their biological replicates were considered outliers and removed from the analysis. T22/control-ratios were converted to log2 and normalized to the average ratio in each experiment. Statistical analysis was made by one-way ANOVA with Tukey–Kramer’s post-hoc test (*p* < 0.05) in Kaleidagraph 4.1.3 (Synergy Software, Reading, PA, USA).

For sand growth, seeds were washed as for soil growth and additionally for 12 h at 10 °C, and then germinated on filter paper soaked with water. Seedlings with fully expanded cotyledons (5–7 days after germination) were transferred to polyethylene boxes (16 × 25 × 8.5 cm) containing a 3 cm layer of a heat-treated (80 °C for 8 h) sand/loam mix (75/25) under conditions as described for soil growth. Genotypes A and F were cultivated side-by-side within each box. The boxes were supplemented with 150 mL of ½ Murashige–Skoog (MS) media, and deionized H_2_O was added for appropriate moisture. After two days of acclimation, each seedling was treated with 0.5 mL of T22 conidiospore suspension (20,000 cfu/mL) or 0.5 mL H_2_O to the root neck. After 8 h of treatment, the seedlings were rapidly (<30 s) harvested by removal of the sand under running water, drying between paper towels, and submerging cut off roots in liquid nitrogen.

### 4.3. Sterile Growth of Sugar Beet Seedlings

For each genotype, approximately 0.5 mL of seeds were extracted from the fruits by manual removal of the pericarp. Seeds were sterilized by treatment with 25 mL 1.8% (*w/v*) NaClO, 5% (*w/v*) Tween 20 for 5 min under slow stirring, and washed twice in 25 mL sterile distilled water for 15 min with slow stirring. Seeds were then placed on the surface of 1/2 Murashige–Skoog (MS) medium [52] with 0.8% (*w/v*) agar in Petri plates, which were sealed with Parafilm. The Petri plates were incubated at 23.5 °C with 50 µmol s^−1^m^−2^ white fluorescent light (Polylux XLRFT8/50 W/840 4000 K; GE Lighting, East Cleveland, OH, USA) for 16 h per day. Sterile seedlings of similar sizes were transplanted after 2–3 days to 12 cm square Petri plates with the same medium, each plate thus containing one seedling of each genotype. Petri dishes were then incubated as above, but in a vertical position. Genotype I was not investigated, because seedlings from extracted and sterilized seeds displayed developmental growth defects, probably due to mechanical damages during extraction.

For growth analyses, treatments of seedlings were made 1 day after transplantation by adding 10–20 cfu of T22 conidiospores to the root neck. Control seedlings were treated with the corresponding volume of sterile water. The plates were incubated at conditions as mentioned above. Results are shown for pooled data from two independent experiments. Another experiment using only genotypes A and F, at lower nutrient level (1/10 MS medium), and using root tip application of the T22 conidiospores, gave similar results as those presented. Box plots were made in Kaleidagraph 4.1. Control and T22-treated seedlings were compared by Student’s t-test (*p* < 0.01).

For transcript analysis of sterile-grown material, genotypes were grown together in the same Petri dishes. The sugar beet seedlings were treated with 40 cfu of germinated T22 conidiospores applied to the root neck 14 days after transplantation.

### 4.4. Colonization Analysis

Sterile-grown seedlings were treated 1 day after transplantation with germinated T22 conidiospores (60–100 cfu/seedling) added to the root neck, and were, after the incubation, stained with lactophenol blue, as described previously [53]. The colonization degree was determined using visual scoring of coded and randomized microscopy images taken at 40 times magnification using an Optiphot−2 microscope (Nikon Corporation, Tokyo, Japan) equipped with a DP70 camera (Olympus Optical, Tokyo, Japan). Scoring was made according to a scale from 0–5, where 0 denotes absence of hyphae, 1 denotes scarce presence of short hyphae, and 5 denotes thick layers of long hyphae covering the root surface.

### 4.5. qRT-PCR

*A. thaliana* marker genes for SA, ET, and JA response pathways [28,29] were used for finding orthologues by two-way BLASTP against the sugar beet genome [24]. Single (non-paralogous) sugar beet genes, for which specific exon-exon border qRT-PCR primer pairs could be designed, were selected. Sugar beet genes encoding glyceraldehyde−3-phosphate dehydrogenase (GAPDH) and isocitrate dehydrogenase (ICDH) were used as references [30,54].

RNA was isolated from roots as previously described [55]. For sterile-grown material, each RNA preparation was made using roots from three seedlings, each one grown in a different Petri dish. Isolated RNA was DNase-treated with the DNA-free DNA Removal Kit (Ambion, Foster City, CA, USA), and cDNA was synthesized using the Maxima First Strand cDNA Synthesis Kit (Thermo Fisher Scientific, Waltham, MA, USA). Transcripts were analyzed by qRT-PCR, principally as in Wallström, Aidemark, Escobar and Rasmusson [55], but using the Maxima SYBR Green qPCR Master Mix (Thermo Fisher Scientific). Annealing temperature was 57 °C for all primer pairs. Each 20 µL PCR reaction contained cDNA corresponding to 0.16 ng total RNA. Transcript levels in T22-treated and control seedlings were compared using Student’s t-test and corrected for multiple testing by the Benjamini–Hochberg method [56] at a false discovery rate of <0.05. For sand-grown material, differences between individual growth boxes were corrected for by mean centering [57] before normalization to the reference genes.

## Figures and Tables

**Table 1:** Primer pairs used.

Gene	Refbeet Code ^1^	*A. thaliana* Orthologue ^2^	Primers (5′−3′) Forward/Reverse	Reference
*GAPDH*	Bv5_107870_ygnn		CATCAAGGCGGAATCAGAAGG/ACGAGCTTTGCGAAGTGGTC	This work
*ICDH*	Bv3u_070630		CACACCAGATGAAGGCCGT/CCCTGAAGACCGTGCCAT	Pin et al. (2010) [30]
*LOX2*	Bv4_072290_uwja	At3g45140	CCAAGATGTTTGATCGGGATCG/ATTCCGTGACACGCTTGATG	This work
*PR1*	Bv9_228910_cfgq	At2g14610	CAAGTAGTGTGGAGAGAATCGG/TGTAATTGCCAGGAGGATCATAA	This work
*PR3*	Bv1_008140_uzgx	At3g12500	AAAGCCAATGTTCGCCTAGC/CAGTAGCCCATCCTCCAGTG	This work
*RAP2–3*	Bv_33470_gcqz	At3g16770	CCGACCTTCTCTCCTCTCATTC/CCGCCCATCCGAGTTGTG	This work

^1^ The *Beta vulgaris* Resource as described at http://bvseq.molgen.mpg.de [24]; ^2^ TAIR10; www.arabidopsis.org.
